# Study Based on the Alliance Between Serum Magnesium Levels and Preterm Labor: An Inclusive Review

**DOI:** 10.7759/cureus.42602

**Published:** 2023-07-28

**Authors:** H S Deeksha, Sandhya Pajai, Srinidhi Cherukuri

**Affiliations:** 1 Obstetrics and Gynaecology, Jawaharlal Nehru Medical College, Datta Meghe Institute of Higher Education and Research, Wardha, IND

**Keywords:** therapeutic interventions, clinical implications, epidemiology, pathogenesis, alliance, serum magnesium levels, preterm labor

## Abstract

Preterm labor, regarded as the onset of labor before 37 weeks of gestation, is a highly prevalent issue in obstetrics with repercussions for neonatal health. This review article presents an in-depth analysis of the alliance between serum magnesium levels and preterm labor. The review explores the physiological roles of magnesium right through pregnancy, including its significance for energy metabolism, smooth muscle contraction, deoxyribonucleic acid (DNA), and protein synthesis. It addresses cellular transport and the homeostasis of magnesium. The pathophysiological processes encompassing inflammation, oxidative stress, calcium regulation, smooth muscle contractility, and neuroendocrine pathways are investigated. The review evaluates epidemiological studies investigating the alliance between serum magnesium levels and preterm labor. The review incorporates an assortment of study varieties, such as observational studies, case-control studies, prospective cohort studies, and meta-analyses. In the course of reviewing the prognostic relevance of serum magnesium levels in premature labor, therapeutic implications involving diagnostic precision, prognostic significance, and therapeutic response assessment have additionally been addressed. Therapeutic interventions targeting magnesium levels, such as magnesium supplementation, tocolytic therapy, and the role of magnesium in antenatal corticosteroid administration, are explored. This review provides an in-depth evaluation of the correlation between serum magnesium levels and preterm labor, stressing its therapeutic significance and repercussions for future research and treatment strategies.

## Introduction and background

Preterm labor, or delivering conception earlier than 37 weeks of gestation, is a significant threat to the world's public health, as preterm infants are seen to have long-term health issues like respiratory distress syndrome, developmental delays, and an increased risk of chronic health conditions later in life. In addition to the heightened instances of newborn morbidity and mortality that this condition has been associated with, it poses substantial challenges for the healthcare system [1]. Many long-term repercussions, which include cerebral palsy, delayed development, poor eyesight, and hearing, are associated with preterm deliveries in survivors [1]. Although multiple states' possible risk variables have been discovered, the precise explanation for preterm labor and delivery continues to be ambiguous. In 50% of worldwide statistical instances, it is anticipated that there are indeed numerous variables at play [2]. Premature rupture of the membranes (PROM), multiple pregnancies, polyhydramnios, hypertensive disorders of pregnancy, infections, cervical incompetence, antepartum hemorrhage, fetal and uterine malformations, anemia, strenuous work, smoking, and other circumstances are among them. PROM is perhaps the most prevalent one [2]. Identifying factors contributing to preterm labor and understanding their underlying mechanisms is essential for developing effective preventive and management strategies.

An essential element called magnesium plays a role in numerous biological processes throughout pregnancy. It is involved in deoxyribonucleic acid (DNA) synthesis, where magnesium acts as a cofactor for DNA polymerization, protein synthesis for enzymatic processes, energy metabolism by the production of ATP, and the regulation of smooth muscle contractility as a calcium channel blocker [3]. Magnesium regulates various physiological procedures and activities as an enzyme cofactor [4]. Magnesium is anticipated to impact the intricate systems involved in the initial and later stages of labor, considering its broad spectrum of functions.

Recent research has investigated the alliance between serum magnesium levels and preterm labor. Alterations in magnesium levels tend to disrupt the delicate harmony of physiological functions, which may harm pregnancy. Magnesium is additionally known to be associated with anti-inflammatory and antioxidant effects, which implies that it might have an essential role in modulating the inflammatory response associated with premature labor [5].

Serum concentrations of magnesium could potentially be assessed as a potential biomarker in assessing the probabilities of preterm labor, in accordance with the cumulative literature. By recognizing expecting women who possess a greater chance for preterm labor and tracking magnesium levels throughout the pregnancy, it may be conceivable to lower maternal and neonatal mortality.

Despite the fact that the hyperlink between serum magnesium levels and preterm labor has already been the focus of several examinations, an in-depth investigation that critically evaluates the information at hand is still essential. This article focuses on the physiological and pathophysiological mechanisms beneath the correlation between serum magnesium levels and preterm labor in the current literature. Understanding the link between magnesium and preterm labor may help healthcare professionals generate decisions with greater understanding while developing preventative and management approaches in this essential field of obstetrics.

## Review

Methodology

To assess the current literature on the alliance between serum magnesium levels and preterm labor, this review paper employed an analytical approach. A comprehensive search strategy was established using appropriate keywords and Medical Subject Headings (MeSH) expressions, and it covered electronic databases such as PubMed, Scopus, and Google Scholar. The search was limited to studies published in English from inception to the present.

Investigation categories corresponding to the inclusion criteria comprised observational, case-control, prospective cohort, and meta-analyses that evaluated any connection between serum magnesium levels and premature labor. Studies focusing on human subjects and reporting relevant outcomes were included. Animal studies, case reports, editorials, and conference abstracts were excluded.

Two unaffiliated reviewers scrutinized the titles and the shortlisted articles' abstracts to assess eligibility. Then, following the predetermined requirements, full-text articles about the study that could potentially be relevant were sourced. The two reviewers' discrepancies had been resolved by dialogue and consensus.

Data extraction was performed using a standardized form, including study characteristics (e.g., study design, sample size, geographic location), participant characteristics (e.g., age, gestational age), measurement of serum magnesium levels, outcome measures, and key findings.

The keywords used for the systematic literature search were "magnesium, "preterm", "delivery", "tocolytic agent", "low birth weight," "preeclampsia," "term," "labor," "corticosteroid," "supplementation", "preterm birth", etc.

The findings from the included studies were synthesized and presented in a narrative format, highlighting key results and the strength of the alliance between serum magnesium levels and preterm labor. The recommendations for future research were provided.

Physiology and functions of magnesium

Role of Magnesium in Pregnancy

Magnesium is a vital mineral that helps contribute to numerous physiological processes that happen to take place throughout pregnancy. Apart from modulating smooth muscle contractility, it also assists in synthesizing proteins, DNA, and energy [1,6,7]. These processes have significance for the fetus' growth and development and for preserving maternal health.

In terms of fetal development, magnesium is essential for proper cellular growth, tissue differentiation, and organ formation [8]. The synthesis of fundamental molecules such as ribonucleic acid (RNA) and proteins, essential for developing the fetal brain, nervous system, and other essential organs, is an additional function it accomplishes [1].

Examining preterm labor is relevant to magnesium's effect on smooth muscle contractility. It aids in regulating the duration of labor and uterine contractions. Imbalances in magnesium levels may influence the excitability of uterine muscles, potentially leading to abnormalities in contractions and the premature onset of labor [1].

In addition, magnesium manages calcium homeostasis and operates as a cofactor for a wide range of enzymatic reactions [4]. Various cellular processes, including muscular contraction and neurotransmitter dissolution, are contingent upon calcium transmission. Magnesium helps maintain the balance of intracellular and extracellular calcium levels, which is essential for proper uterine muscle function during labor [9,10].

It is essential to understand magnesium's multifaceted role throughout pregnancy and how it contributes to preterm labor. Further research is needed to elucidate the specific mechanisms by which magnesium influences the onset and progression of labor, which could pave the way for potential preventive and therapeutic interventions.

Magnesium Homeostasis

Magnesium homeostasis is a tightly regulated process that ensures the balance of magnesium levels in the body (the normal range is between 1.46 and 2.68 mg/dL), including during pregnancy [11]. Maintaining magnesium homeostasis is essential for normal cellular function and overall health. Magnesium accumulates predominantly in two distinct places in the body: intracellular and extracellular [12]. Although extracellular magnesium is found in the bloodstream, intracellular magnesium is heavily correlated with proteins and nucleic acids [13]. Various mechanisms regulate magnesium levels, including intestinal absorption, renal excretion, and exchange with bone [14].

Both passive and active transport processes are essential in the primary pathway of intestinal absorption of magnesium in the small intestine. Renewable excretion heavily influences magnesium levels since the kidneys filter and reabsorb magnesium proportionally to the body's prerequisites [4]. This reabsorption is regulated by hormonal factors, including parathyroid hormone and the active form of vitamin D. In accordance with the body's requirements, exchange with bone acts like a storehouse for discharging or retaining magnesium [9].

During pregnancy, the dynamics of magnesium homeostasis change to accommodate the needs of the developing fetus. Magnesium levels in pregnant women could be impacted by alterations in renal handling and hormonal adjustment, in addition to the higher requirement for magnesium essential for fetal growth and development during pregnancy. Studies have shown that magnesium supplementation prevents preeclampsia and fetal growth restriction and leads to a healthy birth weight [15].

Understanding the intricacies of magnesium homeostasis is crucial for comprehending its alliance with preterm labor. By modifying the biological processes that govern uterine contractions and the duration of labor, perturbations in magnesium homeostasis throughout pregnancy could have a consequence on the likelihood of preterm labor.

Cellular Mechanisms of Magnesium Transport

Magnesium transport across cellular membranes is a complex process involving multiple mechanisms to maintain intracellular magnesium homeostasis [13]. These mechanisms ensure an adequate supply of magnesium for cellular functions while preventing excessive loss or accumulation. Hence, we must properly understand the cellular mechanism followed by magnesium for transport to comprehend its relationship with preterm labor.

Several transporters facilitate the entry of magnesium into cells. The major transporter involved in magnesium uptake is the transient receptor potential melastatin (TRPM) family, specifically TRPM6 and TRPM7 [16,17]. These transporters are expressed in various tissues, including the placenta and uterine smooth muscle cells. They allow magnesium influx into the cells, ensuring its availability for cellular processes [16,17].

On the other hand, magnesium efflux is primarily mediated by the Na+/Mg2+ exchanger (NME) and the magnesium transporter 1 (MagT1). These transporters regulate the extrusion of magnesium from the cells, maintaining intracellular magnesium levels [18].

Furthermore, paracellular transport, which transpires between cells in close proximity, facilitates magnesium traverse within tissues. Tight junction proteins, such as claudins and occludins, regulate paracellular magnesium transport [19]. Dysregulation of these cellular mechanisms of magnesium transport can disrupt intracellular magnesium homeostasis, potentially influencing the risk of preterm labor.

Pathophysiological mechanisms linking magnesium and preterm labor

Inflammation and Oxidative Stress

Inflammation and oxidative stress are two interconnected pathophysiological mechanisms in the alliance between magnesium levels and preterm labor [20]. Magnesium plays a crucial role in modulating inflammation and oxidative stress, and imbalances in magnesium levels can disrupt these processes, potentially contributing to the onset of preterm labor [21].

Preterm labor is considerably impacted by inflammation, which can mainly be demonstrated by the production of pro-inflammatory cytokines, chemokines, and prostaglandins [22]. Magnesium possesses anti-inflammatory properties and can suppress the production of inflammatory mediators [5]. Adequate magnesium levels may help regulate the inflammatory response, reducing the risk of preterm labor.

An additional danger associated with preterm labor is oxidative stress, characterized by an imbalance between the body's defenses against antioxidants and the production of reactive oxygen species (ROS) [23]. Magnesium plays a role in maintaining the state of cellular redox equilibrium and serves as a cofactor for several antioxidant enzymes [21]. Insufficient magnesium levels may impair antioxidant activity, leading to elevated oxidative stress and potential adverse effects on pregnancy outcomes.

Therefore, understanding the interrelationship between magnesium, inflammation, and oxidative stress is essential. Also, it is critical to elucidate the underlying mechanisms linking magnesium levels to preterm labor. For a better understanding of the particular biochemical pathways that are at action and to assess future research and therapeutic approaches that could target these mechanisms, additional research needs to be conducted.

Calcium Regulation

The primary pathophysiological pathway coupling magnesium levels to premature labor has been suggested to revolve around calcium control. It is generally accepted that magnesium and calcium collaborate closely in many different kinds of biological processes, including the contractions of uterine muscles, which are primarily accountable for the start and progression of labor [8].

Magnesium is a natural calcium channel blocker, inhibiting calcium from infiltrating cells [24]. As the literature states, regulating calcium participation is a key component for maintaining the uterine muscle quiescent all throughout pregnancy [25]. This delicate state of equilibrium can be disrupted via imbalances in magnesium levels, which can increase calcium influx and uterine contractility, which might trigger preterm labor [25].

In addition, the link between magnesium and calcium in their regulation of hormone production, especially oxytocin, has been thoroughly recorded. Oxytocin is a key hormone involved in uterine contractions [26]. Magnesium influences the release and action of oxytocin, modulating uterine muscle activity [27].

Understanding the intricate relationship between magnesium and calcium regulation is crucial for comprehending the alliance between magnesium levels and preterm labor. Further research is needed to elucidate the specific molecular mechanisms underlying this interplay and explore potential therapeutic interventions targeting calcium regulation for preventing and managing preterm labor.

Smooth Muscle Contractility

Smooth muscle contractility is a crucial pathophysiological mechanism linking magnesium levels to preterm labor. Magnesium plays a vital role in regulating the excitability and contractility of smooth muscles, including the uterine muscles involved in labor [28].

Magnesium acts as a natural calcium channel blocker, inhibiting calcium influx into smooth muscle cells [24]. By reducing intracellular calcium concentrations, magnesium helps maintain muscle relaxation and prevent excessive uterine contractions [13]. This intricate equilibrium is susceptible to being affected via magnesium level inconsistencies, which may end up in a spike in calcium influx, heightened muscle contractility, and even the prospect of preterm labor.

Furthermore, magnesium interacts with various signaling pathways, such as Direct integrin activation in smooth muscle contraction [29]. It modulates the activity of enzymes, ion channels, and receptors that regulate intracellular calcium levels and the sensitivity of smooth muscle cells to contractile stimuli.

Therefore, understanding the intricate concoction between magnesium and smooth muscle contractility is deemed an essential part of the research for comprehending the alliance between magnesium levels and preterm labor. Further research is needed to unravel the specific molecular mechanisms underlying this interplay and explore potential therapeutic interventions targeting smooth muscle contractility to prevent and manage preterm labor.

Neuroendocrine Pathways

Neuroendocrine pathways significantly influence the alliance between magnesium levels and preterm labor. Magnesium influences several hormonal and neurotransmitter systems involved in the regulation of uterine contractions and the timing of labor.

One key pathway involves the modulation of oxytocin, a hormone crucial for initiating and regulating uterine contractions [27]. Magnesium affects the release and action of oxytocin, influencing the intensity and frequency of uterine contractions [27]. Therefore, any likelihood of disruption in magnesium levels in the body during pregnancy may disrupt the oxytocin signaling pathway, which may lead to aberrant uterine contractibility and preterm labor.

Additionally, it has been reported that magnesium interacts with other neuroendocrine factors, such as prostaglandins and catecholamines, which can influence uterine contractility [30]. Magnesium may modulate these substances' synthesis, release, and activity, further affecting the dynamics of uterine contractions.

Understanding the correlation between magnesium levels and preterm labor necessitates comprehending the intricate relationships between magnesium and neuroendocrine pathways. To prevent and manage preterm labor, further investigation needs to be done to determine the specific mechanisms in action and to explore future research and therapeutic techniques that target neuroendocrine pathways.

Epidemiological studies investigating the alliance

Observational Studies

Observational studies have played a significant role in investigating the alliance between serum magnesium levels and preterm labor. The link between magnesium levels and the possibility of preterm delivery in different demographics has been established by the aforementioned studies.

Various cohort studies have examined the correlation between pregnant women's serum magnesium levels and their probability of preterm labor. Screening pregnancy outcomes and assessing the magnesium level in maternal blood specimens are typical of these investigations. Low maternal serum magnesium levels had been discovered to have a strong correlation with preterm labor, pursuant to research by Okunade et al., Diani et al., Axita et al., and al-Kasseer et al. This could prevent premature birth, provided magnesium levels are maintained appropriately during pregnancy. Furthermore, these findings demonstrate an alliance between low serum magnesium levels and pregnancy-associated issues such as eclampsia, pre-eclampsia, intrauterine growth restriction (IUGR), gestational diabetes, and low birth weight.

Consequently, retaining adequate magnesium levels is a prerequisite for a healthy pregnancy and minimizing the risk of preterm labor [2,31-33]. As a whole, they were able to find that preterm labor is more likely to take place given that serum magnesium levels are decreased [2,31-33].

In addition, case-control studies have compared magnesium levels in women who experienced preterm labor with those who had term deliveries. In a study by Amini Moghaddam et al., a linear classifier algorithm that includes body mass index (BMI), muscle cramps, and serum magnesium levels broadened its capability to forecast premature labor [6]. Comparing patients in term and preterm labor, Ferdous et al. observed significant variations in serum magnesium levels, with low magnesium levels associated with preterm labor [34]. Jenabi et al. noticed that in individuals who experienced premature labor, the serum magnesium levels were substantially lower, the gestational age was substantially reduced, and the birth weight became less [35]. These studies consistently show that women who deliver prematurely have significantly decreased magnesium levels compared to those who continue their pregnancies to term [6,34,35].

Observational investigations provide valuable evidence; however, one needs to understand that they cannot demonstrate causality. The previously observed associations may be altered by biases like confounding variables and reverse causation. Therefore, further research, including randomized controlled trials, must elucidate the causal relationship between magnesium levels and preterm labor.

Case-Control Studies

Case-control research studies have been essential for assisting us in comprehending how serum magnesium levels and premature labor are interrelated. To identify possible variations in magnesium levels among the two groups, these research studies investigate the magnesium levels in women who had term births vs. those who had preterm labor (cases and controls).

Case-control studies have consistently shown that women who deliver prematurely have lower magnesium levels than those with full-term pregnancies. Multiple research investigations have shown a strong correlation between low serum magnesium levels and premature labor. In case-control research, Meena et al. observed that preterm labor patients exhibited lower serum magnesium levels than typical pregnant women reported. Malathi et al. discovered that preterm labor patients showed substantially lower magnesium levels than term labor patients. In cross-sectional case-control research, Okunade et al. concluded that patients with low serum magnesium levels were more likely to have premature labor. According to these results, serum magnesium level assessment might be invaluable for anticipating and avoiding preterm labor, potentially by offering high-risk individuals magnesium supplements [2,36,37]. These findings support the hypothesis that inadequate magnesium levels may increase the risk of preterm labor [38-41].

By examining specific subgroups of women, such as those with known risk factors or medical conditions, case-control studies also help identify vulnerable populations who may be more susceptible to the alliance between magnesium levels and preterm labor.

Notwithstanding the fact that case-control studies provide informative evidence, they have limitations such as recall bias, selection bias, and reliance on retroactive data. Thus, further research is necessary to correspond to the observed relationships and indicate causality, including prospective cohort studies and randomized controlled trials.

Prospective Cohort Studies

Prospective cohort studies have been critical in investigating the alliance between serum magnesium levels and preterm labor. These studies follow a group of women from early pregnancy and assess their magnesium levels over time, subsequently observing the occurrence of preterm labor.

Magnesium levels and the risk of preterm labor typically demonstrate a counterproductive alliance, as demonstrated by prospective cohort studies. The discrepancy in total and ionized magnesium and total calcium levels has been investigated in low-risk pregnancies. Based on research by Arikan et al. in 2000 in Austria, magnesium and calcium levels decline as gestational age advances. However, there was no significant relationship between preterm labor and fluctuations in serum cation levels. As reported by Shaikh et al., low serum magnesium levels have also been linked to problems like intrauterine growth restriction, premature labor, and pregnancy-related toxemia. The findings underscore the physiological significance of low serum magnesium levels in expectant women. A study executed at the University of Benin Teaching Hospital by Enaruna et al. confirms the same observations. Women with lower magnesium levels during pregnancy have been shown to have a higher likelihood of experiencing preterm delivery than those with adequate magnesium levels [42-44].

By collecting data on various potential confounding factors, such as maternal age, gestational age, and medical history, prospective cohort studies allow for adjusting these variables and help establish a more accurate alliance between magnesium levels and preterm labor.

While prospective cohort studies provide valuable evidence, they also have limitations, including the potential for loss of follow-up and the need for large sample sizes to detect significant alliances. Therefore, in order to expand the evidence base and present an extensive understanding of the link between magnesium levels and preterm labor, an amalgamation of prospective cohort studies, case-control studies, and randomized controlled trials needs to be conducted.

Meta-Analyses and Systematic Reviews

Employing data from multiple states, epidemiological investigations, meta-analyses, and systematic reviews have illuminated the alliance between serum magnesium levels and premature labor. These comprehensive analyses help establish a more robust understanding of the relationship and provide a quantitative summary of the evidence.

Meta-analyses have consistently demonstrated a significant inverse relationship between magnesium levels and the risk of preterm labor. Through a systematic review and meta-analysis, Zhang et al.'s findings attempted to identify the relationship between magnesium levels and rates of preterm birth. Ecological, observational, and interventional studies were each included in the study. The researchers observed that higher magnesium levels were associated with a lower risk of preterm birth after evaluating the data that had been made available. They have shown that lower magnesium levels during pregnancy are associated with a higher likelihood of preterm delivery [1].

Systematic reviews comprehensively evaluate the available evidence, identifying strengths, weaknesses, and potential sources of bias across multiple studies. They help assess the consistency and generalizability of the findings, and they inform clinical decision-making and guidelines.

In furtherance of contributing to the body of evidence, the synthesis of data collected from various epidemiological investigations, meta-analyses, and systematic reviews assists in clarifying the alliance between magnesium levels and preterm labor and flags out intriguing subjects for additional investigation.

Clinical implications and predictive value of serum magnesium levels

Diagnostic Accuracy of Serum Magnesium Levels

The diagnostic accuracy of serum magnesium levels in predicting preterm labor has important clinical implications. Determining the predictive value of magnesium levels can aid in identifying high-risk individuals and implementing appropriate interventions to prevent preterm birth. The diagnostic acuity of serum magnesium levels in anticipating preterm labor has been reviewed in many different kinds of research. The sensitivity, specificity, positive predictive value (PPV), and negative predictive value (NPV) for evaluating magnesium levels as a tool for diagnosis have been explored using different cutoff values. The findings suggest that lower serum magnesium levels are associated with an increased risk of preterm labor [1,2,7,36-38,40,41]. However, the diagnostic accuracy of magnesium levels alone is limited. Clinical factors, such as gestational age, maternal risk factors, and additional biomarkers, should be combined to improve the predictive value.

Understanding the diagnostic accuracy of serum magnesium levels can help healthcare providers make informed decisions regarding monitoring, interventions, and management strategies for women at risk of preterm labor.

Prognostic Significance in Preterm Labor

The prognostic significance of serum magnesium levels in preterm labor has important clinical implications. Evaluating the prognostic value of magnesium levels can help predict the outcome of preterm labor and guide management decisions. This research shows that reduced magnesium levels have been associated with an increased risk for undesirable outcomes, including preterm birth, neonatal respiratory distress syndrome, and maternal problems [7].

Assessing the prognostic significance of serum magnesium levels in preterm labor can assist healthcare providers in identifying individuals at higher risk and tailoring appropriate interventions and management strategies. This information can help optimize patient care, improve perinatal outcomes, and guide decisions regarding magnesium supplementation or other interventions to prevent complications associated with preterm labor.

Role in Assessing Treatment Response

Significant clinical connotations arise from assessing serum magnesium levels (the normal level of magnesium in pregnancy is between 0.4 and 0.5 mmol/l) for evaluating treatment performance in premature labor [42]. Evaluating magnesium levels while undergoing medical care may be helpful in assessing the effectiveness of treatment options and determining subsequent treatment strategies. In hospitals, magnesium supplements are frequently administered to treat and avert premature labor. Medical personnel can ensure that the treatment targets have been accomplished and preserved by meticulously tracking serum magnesium levels. The level of magnesium may be routinely evaluated to assess the therapy's effectiveness and determine whether modifications to the dosage are required. Screening serum magnesium levels additionally offers essential data on whether patients consume magnesium supplements. They can point out scenarios in which further interventions or various types of therapy may be required.

The ability to assess treatment response through monitoring serum magnesium levels contributes to individualized patient care and the optimization of therapeutic outcomes in managing preterm labor.

Magnesium as a Potential Biomarker

Serum magnesium levels have emerged as a potential biomarker with clinical implications in preterm labor. The measurement of magnesium levels holds promise for its utility as a predictive and prognostic tool for identifying individuals at risk and monitoring disease progression.

Studies have shown that lower serum magnesium levels are associated with an increased risk of preterm labor and adverse outcomes [2,14,43]. Consequently, identifying particularly vulnerable individuals who would benefit from preventative treatments or periodic surveillance may be simplified by keeping tabs on magnesium levels. Furthermore, magnesium levels could serve as a biomarker for treatment efficacy, permitting healthcare providers to evaluate the effectiveness of interventions and make necessary adjustments.

Further investigation needs to be conducted regarding blood magnesium's capacity as a therapeutic biomarker for evaluating risk, monitoring, and management techniques in preterm labor, especially the straightforwardness of analyzing serum magnesium levels and the link with premature labor.

Therapeutic interventions targeting magnesium levels

Magnesium Supplementation

Magnesium supplementation has been widely explored as a therapeutic intervention to address low serum magnesium levels and potentially prevent or manage preterm labor. The administration of exogenous magnesium can help optimize magnesium levels in pregnant women (the normal range is between 0.4 and 0.5 mmol/l), thereby influencing the risk of preterm birth [B]. Studies have investigated the effects of magnesium supplementation on various outcomes related to preterm labor, including the incidence of preterm birth, neonatal outcomes, and maternal complications. Research demonstrates some potential advantages associated with magnesium supplementation: lessened risk of preterm birth and enhanced neonatal and maternal outcomes [14,45].

To identify the most effective dosage agendas, supplementation durations, and patient selection requirements regarding the prevention and management of preterm labor, a further investigation must be conducted. Comprehending whether magnesium supplements could possibly be administered constructively to maintain adequate magnesium levels provides intriguing opportunities for treatment options that might decrease the likelihood of premature labor.

Tocolytic Therapy

Tocolytic therapy, which aims to inhibit uterine contractions and postpone premature labor, is an additional treatment for improving magnesium levels. Magnesium sulfate is frequently employed as a tocolytic because it effectively relaxes smooth muscle. The beneficial effects of magnesium sulfate as a tocolytic medication in preterm labor have become the focal point of several studies [45-49]. Findings suggest that magnesium sulfate administration can significantly reduce the frequency and intensity of contractions, potentially prolonging pregnancy and improving perinatal outcomes.

Additionally, magnesium sulfate has been investigated for its neuroprotective effects in preterm infants. Evidence supports its use as an antenatal intervention to reduce the risk of neurodevelopmental complications associated with preterm birth [3,50-53]. Though magnesium sulfate tocolytic therapy offers favorable outcomes, it must be considered to evaluate the potential risks to both the mother and the fetus before administering it. To maximize the benefits and limit side effects, careful patient selection, an accurate dose, and continuous surveillance are essential.

Role of Magnesium in Antenatal Corticosteroid Administration

Magnesium serves as a vital underlying nutrient for the effective administration of prenatal corticosteroid therapy, which is frequently administered to enhance fetal lung maturation during circumstances where preterm labor is threatened [54]. Magnesium regulates glucocorticoid receptor function and may influence the response to corticosteroid administration [55].

Studies have investigated the impact of magnesium levels on the efficacy of antenatal corticosteroid therapy. Findings suggest that women with low serum magnesium levels may have a reduced response to corticosteroid treatment, resulting in poorer fetal lung maturation [53]. In circumstances in which low magnesium levels could hinder the effectiveness of corticosteroid therapy, comprehending the significance of magnesium in prenatal corticosteroid distribution may assist with therapeutic decision-making, including dose modifications or switching treatment techniques.

Further research is needed to elucidate the mechanisms underlying the interaction between magnesium and corticosteroid response and determine optimal strategies for ensuring adequate magnesium levels in individuals receiving antenatal corticosteroids.

Future directions and recommendations

Prospective Studies With Larger Sample Sizes

Future investigations with more expansive sample sizes are crucial for broadening our understanding of the correlation between serum magnesium levels and preterm labor. This approach would result in more reliable information since it permits the collection of real-time data and an unbiased evaluation of the correlation between magnesium levels and the outcomes of premature labor. Incorporating different populations would ensure a more powerful implementation, while recruiting additional respondents could improve statistical power and generalizability. Standardized methods for assessing magnesium levels and defining preterm labor outcomes must be established for investigation comparability to render thorough meta-analyses attainable, which may offer an exhaustive assessment of the evidence.

Standardization of Magnesium Assays

The uniformity of magnesium assays should be assigned the greatest importance in any subsequent studies on the connections between serum magnesium levels and premature labor. The precision of result comparisons is compromised by implementing diverse methodologies across different laboratories. Standardized magnesium assays could ensure reproducible and precise readings by combining reference materials, calibration processes, and quality control techniques. Incorporating standardized assays might improve the comparability and authenticity of data, facilitating meta-analyses and systematic reviews for in-depth comprehension. Standardization would make it attainable for doctors to use research results effectively to comprehend and control premature labor.

Mechanistic Studies to Elucidate the Underlying Pathways

Experimental investigations that concentrate on the fundamental procedures must be executed to fully comprehend the relationship between serum magnesium levels and premature labor. These investigations may explain the biological processes involved while shedding light on the specific processes determining how magnesium influences early labor. Understanding might be obtained by researching cellular signaling, gene expression, inflammation, oxidative stress, hormone control, and relationships with components like calcium and progesterone. Adopting revolutionary techniques like omics technologies can make comprehensive molecular analysis feasible. Mechanistic examinations can help investigators find novel therapeutic targets and perhaps create targeted medications that improve magnesium levels, thus improving premature labor outcomes.

Personalized Approaches to Preterm Labor Prevention and Management

Prospective studies in the discipline of preterm labor ought to emphasize customized tactics that consider specific variations in serum magnesium levels and other relevant parameters. The primary objective of personalized medicine is to adjust interventions and management approaches to optimize outcomes while reducing risks. High-risk patients may be recognized using predictive models incorporating serum magnesium levels, biomarkers, clinical characteristics, and genetic variables. Based on individual characteristics, real-time monitoring, point-of-care evaluation, and experimentation with combinations of magnesium supplementation with different treatments could improve outcomes. Employing customized solutions enables individualized interventions and management approaches that adapt to the special requirements of women during pregnancy who are considered at risk for premature labor.

## Conclusions

Last but not least, an extensive body of scientific evidence demonstrates an alliance between low serum magnesium levels and an elevated likelihood of premature labor. Mechanistic investigations have revealed an assortment of mechanisms by which magnesium influences premature labor. Magnesium levels are an intriguing biomarker for risk assessment, outcome prediction, and therapy monitoring, which has profound clinical repercussions. Supplementation and tocolytic therapy are two different modalities of treatment that target magnesium levels and have potential promise in dealing with the symptoms of premature labor. However, additional investigation must be conducted to make up for the limitations. The present research should streamline magnesium assays, enhance sample numbers, perform mechanistic studies, and use personalized approaches to optimize preterm labor's early detection and treatment results.
